# Retraction: MicroRNA-135a alleviates lipid accumulation and inflammation of atherosclerosis through targeting lipoprotein lipase

**DOI:** 10.1039/d1ra90017j

**Published:** 2021-01-22

**Authors:** Laura Fisher

**Affiliations:** Royal Society of Chemistry Thomas Graham House, Science Park, Milton Road Cambridge CB4 0WF UK advances-rsc@rsc.org

## Abstract

Retraction of ‘MicroRNA-135a alleviates lipid accumulation and inflammation of atherosclerosis through targeting lipoprotein lipase’ by Juan Li *et al.*, *RSC Adv.*, 2019, **9**, 28213–28221, DOI: 10.1039/C9RA05176G.

The Royal Society of Chemistry hereby wholly retracts this *RSC Advances* article due to concerns with the reliability of the data. The images in the article were screened by an image integrity expert who found that all the western blot bands are over-contrasted, have no visible background and may not be genuine. Furthermore, the western blots and many other features of the article were found to be unexpectedly similar to western blots and features in a number of other papers with no overlapping authors.

In addition, the paper was analysed by experts who fact-checked the identities of the described nucleotide sequence reagents,^1^ and found errors with the following nucleotide sequence reagents reported in the article. The miR-135a mimic and the si-LPL reagents show no homology to the claimed sequences and, therefore, the results shown in Fig. 3–5 are unreliable.

In addition, the paper was analysed by another expert who found errors with the reagents and nucleotide sequencing reported in the article. Not all of the claimed identities of the sequences reported in the publication are correct for the reported RT-PCR primers, in particular the miR-135a mimic and the si-LPL. These sequences show no homology to the claimed targets and, therefore, the results shown in Fig. 3–5 are unreliable.

The authors were asked to provide the raw data for this article, but did not respond. Given the significance of the concerns about the validity of the data, and the lack of raw data, the findings presented in this paper are not reliable.

The authors have been informed but have not responded to any correspondence regarding the retraction.

Signed: Laura Fisher, Executive Editor, *RSC Advances*

Date: 7^th^ January 2021

## Supplementary Material
